# An Explainable Two-Stage Machine Learning Model for Predicting the Post-Thrombolysis Complications in Stroke Patients: A Multi-Center Study

**DOI:** 10.34133/research.0817

**Published:** 2025-08-19

**Authors:** Hongling Zhu, Qing Ye, Shurui Wang, Hongsen Cai, Mairihaba Maimaiti, Jinsheng Lai, Chuan Qin, Ping Zhang, Yanyan Chen, Qiushi Luo, Hong Wu, Danyang Chen, Shiling Chen, Shudan Zhu, Yuting Lv, Yanxiang Xu, Jian Zhang, Benshan Hu, Yuanxiang Yin, Yan Xie, Dongmei Zhu, Xiaoxing Ming, Zhouping Tang, Hesong Zeng

**Affiliations:** ^1^ Division of Cardiology, Department of Internal Medicine, Tongji Hospital, Tongji Medical College, Huazhong University of Science and Technology, Wuhan, Hubei 430030, P.R. China.; ^2^ Tongji Hospital, Tongji Medical College, Huazhong University of Science and Technology, Wuhan 430030, P.R. China.; ^3^ College of Life Science and Technology, Huazhong University of Science and Technology, Wuhan 430074, P.R. China.; ^4^ Department of Neurology, Tongji Hospital, Tongji Medical College, Huazhong University of Science and Technology, Wuhan 430030, P.R. China.; ^5^ School of Medicine and Health Management, Tongji Medical College, Huazhong University of Science and Technology, Wuhan 430030, P.R. China.; ^6^ Department of Medical Imaging, Yangxin County People’s Hospital, Huangshi, Hubei 435200, P.R. China.; ^7^ Department of Neurology, Xiantao First People’s Hospital Affiliated to Hubei University of Science and Technology, Xiantao, 433000 Hubei, P.R. China.; ^8^ Department of Cardiology, Xiantao First People’s Hospital Affiliated to Hubei University of Science and Technology, Xiantao 433000, Hubei, P.R. China.

## Abstract

Current tools for predicting the thrombolysis risk in patients after stroke exhibit limited event prediction in early post-thrombolysis hemorrhagic events. This highlights an unmet medical need to improve the tools for stroke management. We developed an explainable 2-stage machine learning model for stroke risk stratification to predict the risk of bleeding, composite complications, and all-cause death in patients before and after thrombolysis therapy. The model integrated LightGBM, XGBoost, random forest model (RF), decision tree model (DT), and logistic regression model (LR), and was trained on data from 5,333 patients from Tongji Hospital, achieving improved predictive accuracy in the post-thrombolysis stage compared to the pre-thrombolysis stage. The model exhibited increased area under the curve (AUC) of 0.7581 [95% confidence interval (CI), 0.6955 to 0.8177] and 0.7234 (0.6527 to 0.7909) (bleeding), 0.7625 (0.7324 to 0.7936) and 0.7035 (0.6685 to 0.7392) (composite complications), and 0.9264 (0.8736 to 0.9660) and 0.845 (0.7454 to 0.9375) (death) in post-thrombolysis stage than in pre-thrombolysis stage. External validation using data of 526 patients across 2 different hospitals confirmed the robustness of the model. Key predictors such as temperature, vital signs, and demographic factors were identified. A prototype embedding the best-performing model was constructed. This model enhances thrombolysis risk prediction and supports personalized patient care management, demonstrating its potential for clinical decision support system integration into stroke management strategies.

## Introduction

Stroke is the second leading cause of mortality worldwide and a primary contributor to disability [1]. Ischemic stroke is the main type of stroke, accounting for 85% of strokes, and is characterized by high morbidity, mortality, disability, and recurrence rates [2]. During acute ischemic stroke (AIS), studies estimate that 1.9 million neurons die in the affected area every minute. Thrombosis is the primary pathology of cardiovascular and cerebrovascular diseases that cause death and disability. When a plaque ruptures in blood vessels, it can lead to thrombosis and embolism, ultimately resulting in clinical events such as AIS [2,3]. Therefore, thrombolysis, the administration of thrombolytic agents to dissolve blood clots, is a pivotal treatment strategy endorsed in various guidelines, including those from the American Heart Association and the American Stroke Association, the European Stroke Organization [4], and Chinese Medical Association [5]. Thrombolytic therapy can prevent 30% to 50% of patients with AIS from suffering disabilities [6].

However, despite its efficacy, the thrombolysis rate is limited, particularly in developing and underdeveloped countries, owing to various challenges. First, its effectiveness is highly time sensitive. The European Cooperative Acute Stroke Study has confirmed the effectiveness of thrombolysis and adjusted the thrombolytic time window to 4.5 h [7]. However, delays in the hospitalization process, lack of clear protocols or training, patient awareness, and so on contribute to missed treatment opportunities [8]. Second, the presence of strict contraindications limits the use of thrombolytic agents, often related to underlying health conditions or recent medical history [6]. Absolute contraindications for thrombolytic therapy include abnormalities in cerebral vascular structure, intracranial malignancies, and active hemorrhage. Relative contraindications include age ≥75 years or major surgery within the past 3 weeks [9]. Third, most importantly, the risk of adverse events, particularly bleeding and death, necessitates a precise risk assessment of each patient to made a treatment decision [10]. The probability of symptomatic intracranial hemorrhage after thrombolysis in patients with AIS is 6%, with a mortality rate of 50% to 80% [11]. Thus, clinicians face a challenge to accurately assess the individual risk of complications associated with thrombolysis to make informed treatment decisions.

Various approaches have been proposed to enhance risk assessment and decision-making for thrombolytic therapy. These include statistical analysis of risk factors and the development of prediction models for post-thrombolysis hemorrhagic transformation, as well as prediction scores for post-thrombolysis hemorrhage [12,13]. Cappellari et al. [14] used multivariable logistic regression (LR) analysis to develop the Stroke Treatment and Risk Stratification for Intracerebral Hemorrhage (STARING-SICH) prediction model, which includes 10 indicators, such as systolic blood pressure, age, National Institutes of Health Stroke Scale (NIHSS) score, and blood glucose. This model has a receiver operating characteristic (ROC) area of 0.739 in predicting hemorrhage after stroke thrombolysis. Similarly, Mazya et al. [15] employed an LR model to develop the Symptomatic Intracerebral Hemorrhage Risk Score (SITS-SICH) scoring prediction system, which covers 9 indicators including hypertension, blood glucose, and anticoagulant use. This system assigns scores ranging from 0 to 12 for the risk of hemorrhage from 0.2% to 14.3% after thrombolysis therapy in patients with stroke. However, these methods often show shortcomings in limited event prediction in post-thrombolysis hemorrhage [12,13,16] and limited interpretability.

Machine learning techniques demonstrate marked potential for predicting outcomes and risks in various medical domains. By leveraging large datasets and advanced algorithms, machine learning models can identify patterns and relationships in patient data that may not be apparent to human observers. In the context of thrombolysis for cardiocerebrovascular blockage diseases, machine learning models can potentially provide more accurate risk assessments and aid in personalized treatment decisions [17–19]. Ren et al. [20] built an extreme gradient boosting (XGBoost) machine learning model to analyze hemorrhagic risk in 517 patients undergoing thrombolysis therapy and achieved an area under the curve (AUC) of 0.898 [95% confidence interval (CI), 0.873 to 0.921] in the internal validation cohort. Choi et al. [21] established a neural network model to predict hemorrhagic risk after thrombolysis in 2,028 patients with stroke and achieved an AUC of 0.844. Shen et al. [22] constructed a nomogram model to predict symptomatic intracranial hemorrhage after intravenous thrombolysis in severe white matter lesions.

Nevertheless, most of the reported studies have focused solely on hemorrhagic complications before thrombolysis, without considering other outcomes, such as mortality and complications over time, including those occurring after thrombolysis [21,23,24]. In addition, there is a paucity of research on the 2-stage assessment of risk before and after thrombolysis, despite its crucial role in evaluating the ultimate complications in patients requiring urgent thrombolysis. Moreover, the number of patients with stroke analyzed has been limited, and the data from demographic and laboratory tests, and radiomics have been incomplete [25,26]. For example, Meng et al. [23] built a random forest (RF) model using magnetic resonance imaging radiomics to predict hemorrhage transformation in 71 patients with stroke and obtained an AUC of 0.871. Heo et al. [27] constructed a light gradient boosting machine (LightGBM) model with computed tomography radiomics to predict hemorrhagic transformation risk in patients with stroke undergoing thrombolysis or thrombectomy. In addition, the interpretability and analysis within the black box of the constructed machine learning are lacking or insufficient, and the relationship to clinical usage is lacking [28]. Cui et al. [29] built an XGBoost machine learning model and its prototype clinical decision support system (CDSS) for analyzing the hemorrhagic risk in patients undergoing thrombolysis therapy; however, the included features were limited, and interpretability was not available. Therefore, an urgent need remains for development of a comprehensive, systematic, interpretable, and strongly generalized dynamic model with 2 stages for assessing the thrombolysis risk of stroke and to develop an application system to support the CDSS in practical clinical applications.

In this study, we developed a machine learning-based prediction model to assess multiple complications both before and after thrombolysis in patients with cardio-cerebrovascular occlusive diseases. By utilizing various patient characteristics including demographic information, medical history, laboratory results, imaging findings, and treatment outcomes, we constructed a dynamic, comprehensive, systematic, accurate, interpretable, and generalizable model that can be applied in diverse clinical settings. The results demonstrate that the model can effectively predict the risk of complications at the 2 stages of thrombolysis and enable clinicians to make more informed decisions, exhibiting the potential to improve the management of patients with cardio-cerebrovascular blockage diseases. Thus, the model can help optimize treatment strategies, minimize adverse events, improve patient outcomes, and reduce the mortality and disability rates of cerebrovascular diseases.

## Results

### Characteristics of study population

**Fig. 1.**
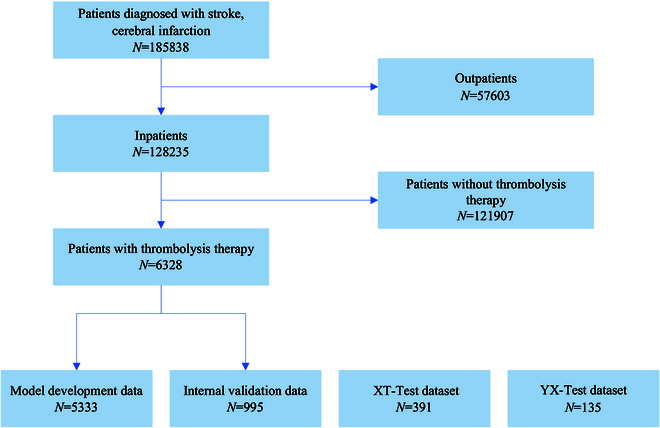
Flowchart of the data included in machine learning process. The XT and YX-Test datasets are 2 independent datasets used for external validation.

The clinical and therapeutic characteristics of the study population are summarized in Fig. 1. A total of 185,838 patients diagnosed with stroke were identified using the big data platform. After excluding outpatients, 128,235 inpatients were included in this study. Among them, 6,328 patients received thrombolysis therapy and were assigned in a ratio of 0.85:0.15 to a model development dataset (Tongji dataset) (*n* = 5,333) and an internal validation dataset (TJ-Test dataset) (*n* = 995) (Table 1). In the Tongji dataset, 303 (5.68%) patients experienced bleeding, 1,508 (28.28%) had composite complications, and 65 (1.22%) died. The nonreplicated TJ-Test dataset of 995 patients revealed bleeding in 65 patients (6.53 %), composite complications in 181 patients (18.19 %), and death in 11 patients (1.11 %). Patients in the Tongji dataset had a median age of 67 years (59 to 76) with a male proportion of 68.69%. Similarly, patients in the TJ-Test dataset exhibited a comparable age distribution, with a median age of 66 years (58 to 74) and a male proportion of 67.74%. The Tongji dataset also exhibited a high occurrence of comorbidities, including hypertension (12.06%), diabetes (10.78%), familial clustering disease (28.91%), high allergy history (34.67%), smoking history (34.24%), and drinking history (23.23%). The TJ-Test dataset exhibited a consistent distribution. The most commonly administered thrombolytic agents in the Tongji dataset were urokinase (27.64%), fibrinolysin (66.40%), and alteplase (8.31%). In contrast, in the TJ-Test dataset, the thrombolytic agents used were urokinase (46.83%), fibrinolysin (43.92%), and alteplase (9.25%). Detailed information on the external validation datasets (YX and XT datasets) is provided in Table S1.

**Table 1.** Clinical features of the patients in Tongji and TJ-Test datasets. Data are shown as *n* (%), or *n*/*N* (%), and medium (IQR), where *N* is the total number of patients with available data.

Characteristic	Tongji dataset (*n* = 5,333)	TJ-Test dataset (*n* = 995)
Demographic information		
Age (years)	67 (59–76)	66 (58–74)
Sex (male)	3,663 (68.69%)	674 (67.74%)
Comorbidities		
Coronary heart disease	99 (1.89%)	20 (2.01%)
High blood pressure	643 (12.06%)	123 (12.36%)
Diabetes	575 (10.78%)	102 (10.25%)
Hyperlipidemia	128 (2.4%)	17 (1.71%)
Cerebrovascular diseases	167 (3.13%)	14 (1.41%)
Pregnant history	1,409 (26.42%)	234 (23.52%)
Surgical history	870 (16.31%)	221 (22.21%)
Familial clustering disease	1,542 (28.91%)	547 (54.97%)
Allergy history	1,849 (34.67%)	227 (22.81%)
Smoking history	1,826 (34.24%)	264 (26.53%)
Drinking history	1,239 (23.23%)	171 (17.19%)
Timing (min)		
Onset time	144 (48–720)	168 (48–1,440)
In-hospital delay time	20.77 (15.04–68.99)	39.45 (16.78–107.88)
Thrombolytic agent		
Urokinase	1,474 (27.64%)	466 (46.83%)
Fibrinolysin	3,541 (66.40%)	437 (43.92%)
Alteplase	443 (8.31%)	92 (9.25%)
Reteplase	2 (0.04%)	0
Recombinant streptokinase	1 (0.02%)	0
Pre-thrombolysis vital signs		
Diastolic blood pressure (mm Hg)	84 (76–92)	83.19 (76–90)
Systolic blood pressure (mm Hg)	144 (129–158)	142.04 (129–154)
Respiratory rate (breaths/min)	20 (18–20)	20 (18–20)
Temperature (°C)	36.5 (36.3–36.6)	36.5 (36.3–36.6)
Post-thrombolysis vital signs		
Diastolic blood pressure (mm Hg)	78 (73.96–83)	76.67 (73–80.92)
Systolic blood pressure (mm Hg)	132 (122–143)	131 (121–144)
Respiratory rate (breaths/min)	19 (18–20)	20 (18–20)
Temperature (°C)	36.5 (36.3–36.7)	36.5 (36.3–36.6)
Medication use during hospitalization		
ACEI	401 (7.52%)	98 (9.85%)
ARB	451 (8.46%)	116 (11.66%)
β Blocker	834 (15.64%)	162 (16.28%)
Statin	1,004 (18.83%)	191 (19.20%)
Clopidogrel	168 (3.15%)	36 (3.62%)
Aspirin	54 (1.01%)	13 (1.31%)
Outcomes		
Bleeding	303 (5.68%)	65 (6.53%)
Composite complications	1,508 (28.28%)	181 (18.19%)
Death	65 (1.22%)	11 (1.11%)

ACEI, angiotensin-converting enzyme inhibitor; ARB angiotensin receptor blocker

In the bleeding group pre-thrombolysis, the LR model achieved the highest AUC of 0.7234 (95% CI, 0.6527 to 0.7909), followed by the RF model with AUC of 0.6958 (95% CI, 0.6269 to 0.7607) and XGBoost model with AUC of 0.6568 (95% CI, 0.5843 to 0.7351). As for the bleeding post-thrombolysis group, the RF [AUC, 0.7581 (95% CI, 0.6955, 0.8177)], XGBoost [AUC 0.7553 (95% CI, 0.6837 to 0.8197)], LightGBM [AUC, 0.7484 (95% CI, 0.6769 to 0.8137)], and LR [AUC, 0.7469 (95% CI, 0.6767 to 0.8074)] models showed a similar AUC. The decision tree (DT) model [AUC, 0.659 (95% CI, 0.5780 to 0.7336)] performed the least well. Thus, in the bleeding group, we chose the LR and RF models as the best-performing models for pre- and post-thrombolysis, respectively.

For the prediction of composite complications, the RF model performed with the highest AUC of 0.7035 (95% CI, 0.6685 to 0.7392) for pre-thrombolysis and LightGBM with the highest AUC of 0.7625 (95% CI, 0.7324 to 0.7936) for post-thrombolysis. The RF model also showed the highest accuracy of 0.7291 (95% CI, 0.7038 to 0.7545), precision of 0.5244 (95% CI, 0.4640 to 0.5787), and specificity of 0.8404 (95% CI, 0.8153 to 0.8646) compared with the other 4 models for pre-thrombolysis. Therefore, we chose the RF model for pre-thrombolysis and LightGBM model for post-thrombolysis as the best-performing models in the composite complication group.

**Fig. 2.**
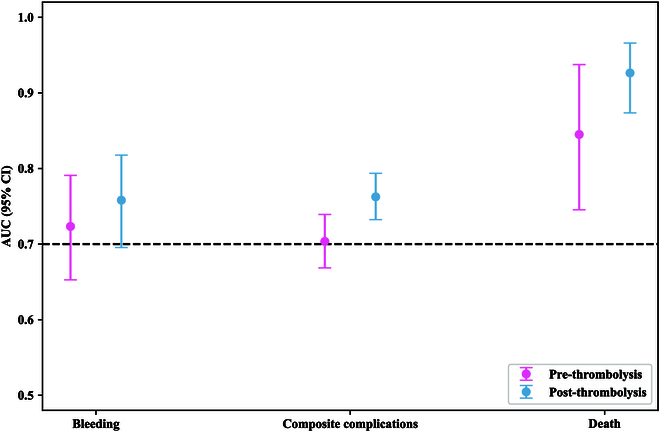
The AUCs of the best-performed model for bleeding, composite complications, and death pre- and post-thrombolysis on the Tongji dataset. AUC, area under the curve; CI, confidence interval.

**Fig. 3.**
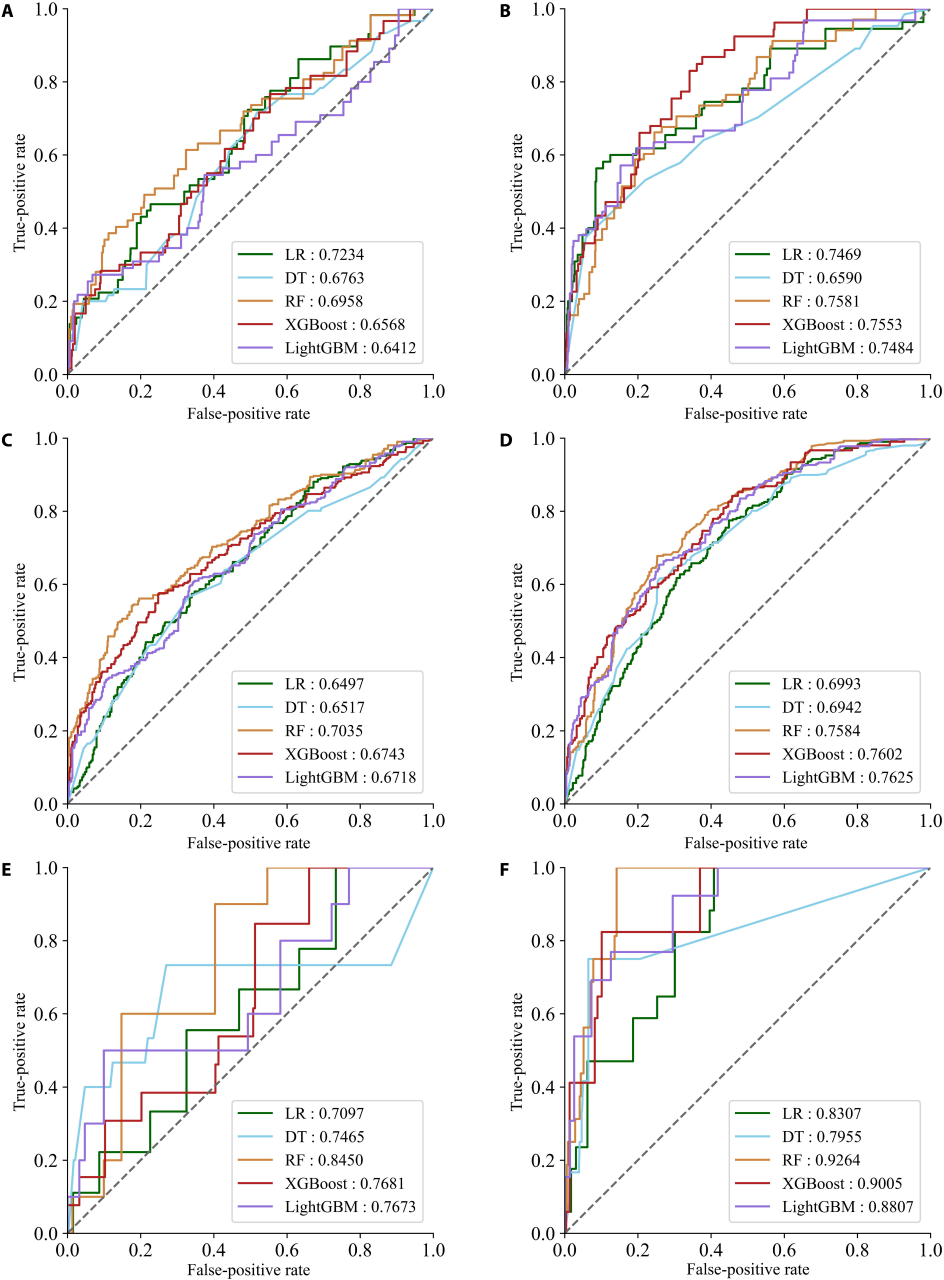
Area under the ROC curves of the 5 models for bleeding, compound complications, and death pre-thrombolysis and post-thrombolysis on the Tongji dataset. Bleeding pre-thrombolysis (A) and post-thrombolysis (B), composite complication pre-thrombolysis (C) and post-thrombolysis (D), and death pre-thrombolysis (E) and post-thrombolysis (F). LR, logistic regression; DT, decision tree; RF, random forest; XGBoost, extreme gradient boosting; LightGBM, light gradient boosting machine.

In terms of mortality prediction at the pre-thrombolysis stage, the RF model showed superior performance with the highest AUC of 0.845 (95% CI, 0.7454 to 0.9375) compared with those of XGBoost [AUC, 0.7681 (95% CI, 0.6345 to 0.8891)], LightGBM [AUC, 0.7673 (95% CI, 0.5868 to 0.9307)], DT (AUC, 0.7645 (95% CI, 0.5244 to 0.9278)], and LR [AUC, 0.7097 (95% CI, 0.5597 to 0.8514)]. The RF also achieved an accuracy of 0.8351 (95% CI, 0.8135 to 0.8566), a sensitivity of 0.6923 (95% CI, 0.4375 to 0.9375), and a specificity of 0.8371 (95% CI, 0.8159 to 0.8585) in mortality prediction for the pre-thrombolysis stage. The RF model showed the highest AUC of 0.9264 (95% CI, 0.8736 to 0.9660) compared with those of XGBoost [AUC, 0.9005 (95% CI, 0.8209 to 0.9630)], LightGBM [AUC, 0.8807 (95% CI, 0.8032 to 0.9379)], LR [AUC, 0.8307 (95% CI, 0.7420 to 0.9089)], and DT [AUC, 0.7955 (95% CI, 0.6257 to 0.9344)] for the post-thrombolysis stage. The RF model achieved a high accuracy of 0.8491 (95% CI, 0.8266 to 0.8697), sensitivity of 0.9286 (95% CI, 0.75, 1), and specificity of 0.8480 (95% CI, 0.8256 to 0.8680). Thus, the RF model was ranked as the best-performing model in the death group before and after thrombolysis (Table 2, Table S2, and Fig. 1), demonstrating that the post-thrombolysis model performed better with higher AUCs, accuracy, and specificity than the pre-thrombolysis model (Figs. 2 and 3). Furthermore, we investigated the recall (sensitivity) curves and found that the models exhibited high sensitivity at a lower threshold (Fig. S2). Calibration curves of the best-performing mode (Fig. S3) demonstrated a relatively stable performance on the Tongji cohort, but exhibited some degree of overfitting indicating room for improvement in its overall calibration ability.

**Table 2.** Model performance of bleeding, composite complications, and death with the highest AUC on the Tongji dataset

Group	Model	AUC (95% CI)	Accuracy (95% CI)	Precision (95% CI)	Specificity (95% CI)	Sensitivity (95% CI)	F1 (95% CI)
Bleeding							
Pre	LR	0.7234 (0.6527–0.7909)	0.7048 (0.6767–0.7320)	0.1107 (0.0800–0.1472)	0.7119 (0.6832–0.7388)	0.5915 (0.4643–0.7167)	0.1866 (0.1362–0.2414)
Post	RF	0.7581 (0.6955–0.8177)	0.9157 (0.8978–0.9316)	0.2647 (0.1666–0.3696)	0.9533 (0.9396–0.9665)	0.2797 (0.1764–0.3913)	0.2731 (0.1724–0.3670)
Composite complications							
Pre	RF	0.7035 (0.6685–0.7392)	0.7291 (0.7038–0.7545)	0.5244 (0.4640–0.5787)	0.8404 (0.8153–0.8646)	0.4466 (0.3947–0.5000)	0.4828 (0.4340–0.5271)
Post	LightGBM	0.7625 (0.7324,0.7936)	0.7441 (0.7170–0.7685)	0.5543 (0.4941–0.6157)	0.8524 (0.8278–0.8777)	0.4660 (0.4080–0.5232)	0.5065 (0.4544–0.5550)
Death							
Pre	RF	0.8450 (0.7454–0.9375)	0.8351 (0.8135–0.8566)	0.0479 (0.0220–0.0833)	0.8371 (0.8159–0.8585)	0.6923 (0.4375–0.9375)	0.0892 (0.0421–0.1500)
Post	RF	0.9264 (0.8736–0.9660)	0.8491 (0.8266–0.8697)	0.0699 (0.0341–0.1124)	0.8480 (0.8256–0.8680)	0.9286 (0.75–1)	0.1297 (0.0658–0.2010)

AUC, area under the curve; CI, confidence interval; LR, logistic regression; RF, random forest; LightGBM, light gradient boosting machine

Next, we examined the best-performing model for both pre- and post-thrombolysis using the internal validation TJ-Test dataset. The LR model exhibited an AUC of 0.7286, accuracy of 0.9065, precision of 0.2742, specificity of 0.9516, sensitivity of 0.2615, and F1 of 0.2677 for pre-thrombolysis. Meanwhile, the RF model showed an increase in AUC to 0.825, accuracy of 0.9327, and specificity of 0.9968 for post-thrombolysis, with a slightly decreased precision of 0.25 for post-thrombolysis. However, the F1 score and sensitivity post-thrombolysis were 0.029 and 0.0154, with an obvious decrease compared to those pre-thrombolysis, indicating that the RF model showed higher specificity and accuracy for detecting patients in the bleeding group post-thrombolysis than the LR model for pre-thrombolysis.

In the composite complication group, the LightGBM model showed an AUC of 0.6862, accuracy of 0.8151, precision of 0.4681, sensitivity of 0.1215, and F1 of 0.193, all of which were higher than those of pre-thrombolysis using the RF model. The specificity was similar between pre-thrombolysis (0.9828) and post-thrombolysis (0.9693), indicating that the LightGBM model had superior performance in assessing patients post-thrombolysis compared to the RF model for pre-thrombolysis.

Similar results were observed in the death prediction group. The RF model had an increased AUC of 0.8615, accuracy of 0.7588, precision of 0.0364, specificity of 0.7581, sensitivity of 0.8182, and F1 of 0.0698 for post-thrombolysis compared to the RF model for the assessment of pre-thrombolysis (AUC, 0.6864; accuracy, 0.6312; precision, 0.0215; specificity, 0.6301; sensitivity, 0.7273; F1, 0.0418) (Table 3).

**Table 3.** Result of best-performing model for bleeding, compound complications, and death pre-thrombolysis and post-thrombolysis on the TJ-Test dataset

Group	Model	AUC	Accuracy	Precision	Specificity	Sensitivity	F1
Bleeding							
Pre	LR	0.7286	0.9065	0.2742	0.9516	0.2615	0.2677
Post	RF	0.825	0.9327	0.25	0.9968	0.0154	0.029
Composite complications							
Pre	RF	0.6174	0.8131	0.3913	0.9828	0.0497	0.0882
Post	LightGBM	0.6862	0.8151	0.4681	0.9693	0.1215	0.193
Death							
Pre	RF	0.6864	0.6312	0.0215	0.6301	0.7273	0.0418
Post	RF	0.8615	0.7588	0.0364	0.7581	0.8182	0.0698

The above analysis using the internal validation of TJ-Test dataset revealed that the model performance was consistently superior in the assessment of post-thrombolysis compared with pre-thrombolysis in terms of AUC and accuracy across all 3 outcomes. Similar trends were observed when using external validation datasets, where the AUCs of the models remained higher for the assessment of post-thrombolysis than for pre-thrombolysis in most cases (Table S3 and Fig. S4). We examined all the 6,328 samples in the Tongji cohort (model development and internal validation datasets) and analyzed their NIHSS scores. We identified 137 patients with NIHSS score of 7 (3, 12). We set a score of 0 to 3 as low risk, and 3 to 14 as high risk. The AUC of the NIHSS was 0.5233 for classifying bleeding and 0.3731 for classifying composite complications, which were lower than those of our machine learning models. Patients with NIHSS scores did not belong to the death group; therefore, the AUC for mortality could not be calculated. These indicate that our models can not only function as an artificial intelligence (AI)-enabled prediction model for 3 different clinical outcomes of thrombolysis but also serve as a dynamic tool for the assessment of patients before and after thrombolysis with improved performance.

### Explainability of the model

**Fig. 4.**
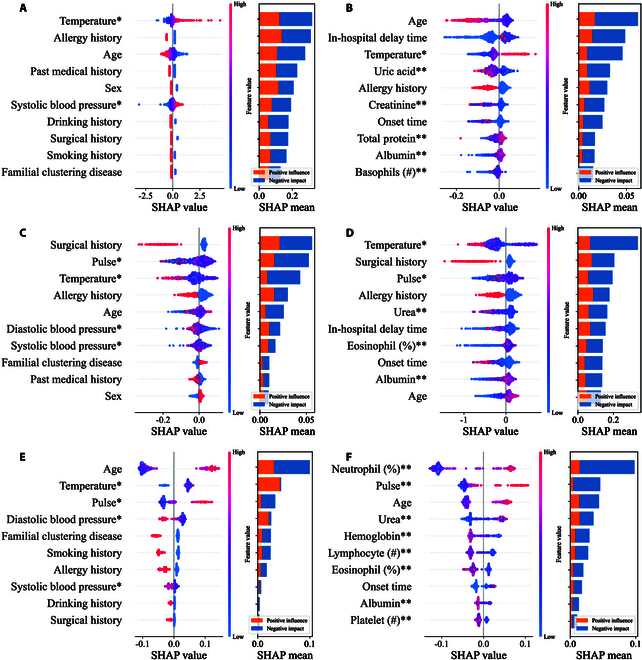
SHAP analysis of the best-performed model of bleeding, composite complications, and death pre-thrombolysis and post-thrombolysis on Tongji dataset. Bleeding pre-thrombolysis (A) and post-thrombolysis (B), composite complication pre-thrombolysis (C) and post-thrombolysis (D), and death pre-thrombolysis (E) and post-thrombolysis (F). *, pre-thrombolysis; **, post-thrombolysis.

As shown in Fig. 4, Shapley additive explanations (SHAP) analysis was used to indicate the 10 most important predictors of bleeding, composite complications, and death pre- and post-thrombolysis, with red and purple representing positive and negative values, respectively. The SHAP values show the importance of these features to the outcomes, and a higher SHAP value indicates a higher contribution to the outcome.

The pre-thrombolysis temperature was the most important predictor, followed by allergy history and age in pre-thrombolysis stage. Age, in-hospital delay time, and pre-thrombolysis temperature were the 3 most important predictors of the post-thrombolysis bleeding group. This result highlights the importance of the time window from disease onset to treatment in the assessment of bleeding. Pre-thrombolysis temperature and age reflect initial patient’s condition and infection level, suggesting a crucial role in bleeding outcome prediction.

Among the composite complications, surgical history, pre-thrombolysis pulse, and temperature were the top predictors at the pre-thrombolysis stage, followed by diastolic blood pressure, age, and systolic blood pressure. Post-thrombolysis, pre-thrombolysis temperature, surgical history, pre-thrombolysis pulse, in-hospital delay time, post-thrombolysis uric acid, and eosinophil count (%) were important predictors. This indicates that the patient’s vital signs, including surgical history, play an important role during the pre-thrombolysis period, and the initial patient condition, particularly temperature and pulse, are important in predicting post-thrombolysis complications.

In the death group, age emerged as the most important predictor at the pre-thrombolysis stage, followed by temperature, pulse, and diastolic blood pressure. At the post-thrombolysis stage, neutrophil (%), pulse, age, and urea level were significant predictors. This indicates the impact of infection and functional status of vital organs on mortality.

In addition, radar plots were also used to indicate the 8 most important predictors of the 3 outcomes at the 2 stages, as shown in Fig. S5. This shows great similarity and consistency to those highlighted by the SHAP analysis and is especially identical in the death group. This further enhanced the interpretability of the model.

### Implementation of CDSS

**Fig. 5.**
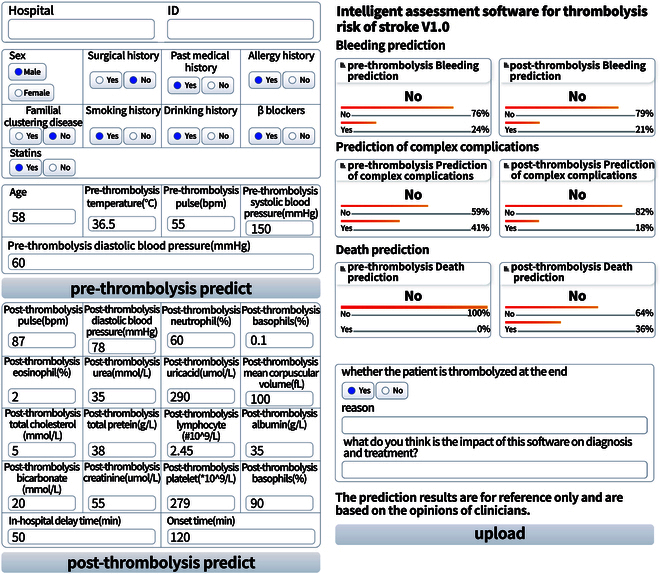
CDSS interface of the prototype system of the prediction models.

We integrated the best-performing models (LR for pre-thrombolysis and RF for post-thrombolysis in the bleeding group, RF for pre-thrombolysis and LightGBM for post-thrombolysis in the composite complication group, and RF for both pre- and post-thrombolysis in the death group) into a prototype system. This system can predict the risk of bleeding, composite complications, and death as the core task, and serves as a valuable tool for decision-making by clinicians. Its interface is shown in Fig. 5 and includes the patients’ general hospital admission details, form of the input parameters, and results. Specifically, the patients’ general hospital admission details include the hospital and patient ID, and the hospital can be selected through a dropdown menu listing the major hospitals in China. The hospital name can also be input manually. The main part of the CDSS prototype, where the parameters can be typed, is as follows. Each parameter is accompanied by detailed unit annotations, and general and medical history information are presented in the form of multiple-choice questions. This setup can standardize the input and avoid erroneous and inconsistent entries. The interface displayed on the right groups the outcomes by complications and further subcategorizes them into pre- and post-thrombolysis, making the results clear at a glance. Thus, the system interface has been designed to be user-friendly and intuitive, making it easy for clinicians to input relevant parameters and obtain risk analysis results within 1 s.

Doctors can include relevant parameters such as vital signs, medical history, and thrombolysis details on the form provided. Then, by simply clicking the button, the pre-thrombolysis predict and post-thrombolysis predict, the system generates the risk analysis results. The results are presented in a quantified format, displaying the probability values for each complication (bleeding, composite complications, and death) during the pre- and post-thrombolysis stages. This prototype system streamlines the risk-prediction process for thrombolytic complications, providing clinicians with valuable insights for optimizing patient care and treatment decisions. By presenting the probability values for bleeding, composite complications, and death, the system allows clinicians to better understand the potential risks associated with thrombolysis and make more informed treatment decisions. The CDSS software has been deployed first in the Yangxin County People’s Hospital in Huangshi, China. Further prospective real-world clinical trials should be conducted. Detailed information is available at http://81.70.243.91:8001/.

## Discussion

In our study, using large-scale retrospective real-world clinical databases from multiple centers, we developed an explainable 2-stage prediction model using AI-enabled machine learning techniques to predict the risk of bleeding, composite complications, and death both pre- and post-thrombolysis, with strong interpretability consistent with clinical practice. Furthermore, we established a CDSS prototype system that implemented the prediction model, thereby enhancing the clinical practice.

We evaluated 5 different machine learning-based prediction models in predicting the risk of bleeding, composite complications, and death pre- and post-thrombolysis and found that the models exhibit an increased accuracy in risk assessment for the post-thrombolysis stage compared to the pre-thrombolysis stage, and an increased AUCs compared to that of the clinical NIHSS score assessment model. Simultaneously, the thrombolysis complication prediction in our study included bleeding, composite complications, and death, which represents a comprehensive and wide range of complications in thrombolysis therapy. Composite complications represent the condition of the heart and blood vessels as well as the functional status of vital organs, such as the heart. This complication prediction provides a scientific basis for early intervention in patients with complex and changing conditions, as well as the deterioration of vital organ functions [7,30]. The 2-stage and generable characteristics enable the model to perform efficiently and help clinicians to diagnose patient complications and provide timely treatment. We further validated the model performance using multiple datasets, including internal and external datasets from 2 different hospitals, based on randomly collected electronic health record (EHR) data, strengthening its generalizability and applicability. Consistent performance across these datasets underscores the robustness of the model.

In predictive models for thrombolysis-related outcomes (bleeding, composite complications, and death), sensitivity (true-positive rate) has critical clinical significance, especially when prioritizing the identification of high-risk patients. Extremely low sensitivity values pose the risk of under-detecting patients likely to experience adverse events, which could ultimately result in delayed interventions or overlooked preventive measures [31]. In our study, the highest sensitivity of the models ranged from 0.5915 (95% CI, 0.4643 to 0.7167) (LR in the bleeding pre-thrombolysis stage) to 0.9286 (95% CI, 0.75, 1) (RF in the death post-thrombolysis stage), which was lower than the specificity ranging from 0.7119 (95% CI, 0.6832 to 0.7388) to 0.9943 (95% CI, 0.9895 to 0.9981), respectively. To address this imbalance, we investigated the recall threshold curves and found that lowering the threshold could enhance sensitivity [32]. In practical clinical applications, it is necessary to adjust the threshold according to the complexity of the patient’s condition to improve sensitivity and reduce the false-negative rate, even at the cost of modest specificity trade-offs [33]. Calibration curves assessing prediction-reality alignment are essential for quantifying the net clinical benefit across sensitivity-driven risk thresholds [34]. Therefore, from a clinical perspective, our model should prioritize sensitivity in predicting life-threatening post-thrombolytic complications.

Our model further highlights the key predictors for assessing the risk of bleeding, complications, and death during thrombolytic therapy. Notably, pre-thrombolysis temperature and age were consistent and significant predictors of bleeding, composite complications, and death, highlighting the importance of the initial patient status across various organ systems. Abnormal body temperature can induce brain injury [35], circadian changes [36], metabolic disorders, immune suppression, and more, impacting nearly every physiological function of the body [31]. Additionally, for composite complications, vital signs like surgical history, pulse, and temperature are crucial indicators, followed by blood pressure pre-thrombolysis and in-hospital delay time and onset time post-thrombolysis. These findings underscores the importance of both the patient’s initial condition and timeliness of treatment in assessing composite complication risk. Age remained a significant predictor of death, both pre- and post-thrombolysis. Infection markers such as neutrophil percentage and organ function indicators such as pulse and urea levels are important for the assessment of post-thrombolysis death.

Ping et al. [37] developed an RF model to identify increased systolic pressure, blood glucose, and cholesterol in patients with poor outcomes with a higher modified Rankin score (mRs) of 3 compared to those with good outcomes with mRs < 2. Lyu et al. [38] identified systolic blood pressure, lymphocyte percentage, and platelet to lymphocyte ratio as the independent predictors of neurological deterioration in patients with AIS after thrombolysis therapy using univariate and multivariate LR analysis. Soni et al. [39] identified age as a predictor of bleeding and poor prognosis in patients with stroke after thrombolysis therapy. Iosa et al. [40] identified age using an artificial neural network to be a common factor influencing neurorehabilitation outcomes of patients with stroke treated with or without thrombolysis therapy. Peripheral white blood cell counts, including neutrophils, serving as convenient markers of systemic inflammation, are believed to undermine the stability of atherosclerotic plaques [41]. All our findings, in accordance with scientific literature, underscore the need for timely interventions, monitoring of patient conditions, and consideration of individual patient characteristics in clinical decision-making.

We created an easy-to-implement CDSS integrated with our top-performing models, which can help identify patients at risk of developing certain complications, including bleeding, composite complications, and death, enabling early intervention and prevention. As reported in various studies [42,43], the integration of CDSS into EHRs enables real-time access to diagnostic suggestions, tailoring treatment plans, and preventative measures based on a comprehensive analysis of patient data. This approach ensures that clinical decisions are informed by the most up-to-date and relevant information, enhancing their efficiency, accuracy, and patient-centeredness [44]. Recognized as a crucial step in advancing clinical practice, this approach allows for a seamless integration of patient information and medical knowledge, providing physicians with invaluable support in clinical decision-making [45]. The ease of use is a key advantage of this system. The interface is designed to be straightforward and easy to navigate, allowing clinicians to quickly input the necessary parameters and obtain results. This is particularly important in emergency situations, where prompt decision-making by clinicians is crucial [46]. The broad applicability of the system is another key advantage, as it can be used in various clinical settings where thrombolysis is performed. The system models were trained on a large dataset, ensuring reliable predictions. Additionally, the system can be used for educational purposes to train new clinicians on the risks and benefits of thrombolysis. With advances in internet technology, we foresee that this system has a particularly promising use in telemedicine, especially for centers and hospitals in rural areas of the world where experienced doctors are scarce [47]. In conclusion, our proposed prototype system is a valuable tool for predicting the risk of bleeding, composite complications, and death related to thrombolysis. Its user-friendly interface, ease of use, broad applicability, and potential impact make it a promising addition to clinical decision-making.

### Limitations

Although our study represents a significant step forward, some limitations should be addressed in future studies. First, as a retrospective study, it is prone to design biases (e.g., unmeasured confounding factors). Second, the lack of clinical scales for composite complications and death limits the ability to compare the model performance with established benchmarks. Although scales for hemorrhage assessment exist, they are rarely assessed due to high costs, and as a retrospective study, it is difficult to complete scales such as the NIHSS and SITS-SICH. Third, despite the model achieving high specificity, the relatively low sensitivity for complication prediction highlights the need for further refinement to reduce false negatives in high-risk populations, which will be further emphasized in our next study. Fourth, the performance of the model on the external validation datasets may be affected by small cohorts (limited to 526 cases from XT/YX centers), data quality issues, and the limited scale of the hospitals. Because the outcomes and a few clinical features are derived from textual indicators, the quality of EHRs is critical. This inconsistent case quality results in a performance that falls short of expectations for external datasets. Prospective validation and further validation on larger datasets will be an important aim of our next study to demonstrate the robustness and generalizability of the model [48,49]. Considering issues related to patient safety and ethics, future studies may employ laboratory experiments to address these issues.

## Conclusion

We developed an explainable 2-stage prediction model using AI-enabled machine learning to predict the risk of bleeding, composite complications, and death both pre- and post-thrombolysis, with strong interpretability consistent with clinical practice. Furthermore, we established a CDSS prototype system that implemented this prediction model, which shows promising potential in enabling the effective reduction of examinations and facilitates early intervention and prevention, thus providing invaluable support to clinical decision-making for patients with cardio-cerebrovascular blockage diseases.

## Methods

### Patients and data sources

We retrieved EHR data from hospitalized patients diagnosed with stroke according to the International Statistical Classification of Diseases and Related Health Problems, 10th Revision (ICD-10), at 3 campuses (Main Campus, Optical Valley Campus, and Sino-French New City Campus) of the Tongji Hospital of Huazhong University of Science and Technology, Wuhan, China, from March 2003 to August 2023. The dataset was managed using a dynamically updated big data intelligence platform (https://datacenter.tjh.com.cn/). It comprised of deidentified and comprehensive EHR data that were meticulously structured and processed. Patient data were extracted from various EHR systems integrated into a unified platform, including electronic medical records, radiation information systems, laboratory information systems, ultrasound systems, and electrocardiogram systems. All patients on the platform were distinguished and regulated through a unique serial number (SN), a 32-digit number automatically generated by the platform corresponding to the patient’s Country Resident ID card. This effectively prevented the same patient admitted to hospitals using different IDs from entering the same training/testing dataset. We collected demographic information, chief complaints, medical history, and examinations and test results for both the in-hospital pre-thrombolysis stage and initial post-thrombolysis stage. Patients <18 years of age, patients with severe liver or kidney dysfunction, malignant tumors, sepsis, and severe mental illness that hindered treatment cooperation, and patients lacking conclusive outcomes were excluded. Thrombolytic therapy was defined as the administration of thrombolytic drugs, including urokinase, fibrinolysin, alteplase, reteplase, tenecteplase, and recombinant streptokinase.

This study was approved by the ethics committee of Tongji Hospital of Huazhong University of Science and Technology (approval number TJ-IRB202406025). The requirement for written informed consent was waived due to the retrospective design of the study. The internal validation dataset (TJ dataset) consisted of 995 nonreplicated patients (distinguished from duplicates through the unique SN) from Tongji Hospital, which were randomly split from the included datasets at a ratio of 15%. For external validation, we included 135 patients (hospitalized between 2020 July 23 and 2023 August 24) and 391 patients (hospitalized between 2017 November 3 and 2023 November 2) from Yangxin County People’s Hospital in Huangshi, China (YX dataset) and Xiantao First People’s Hospital in Xiantao, China (XT dataset). The data collection team was responsible for sample collection and anonymization, and the algorithm development team received anonymized data with only patient identification number, age, sex, and other clinical information for subsequent algorithm development and validation. All modeling work was conducted on the Tongji Hospital Big Data Intelligence Platform, which prevents data leakage and promotes data security.

### Outcomes

The outcomes were classified according to the European Stroke Organisation guidelines [4]. The main risks of thrombolysis include intracranial hemorrhage, cardiac and vascular function status, and all-cause mortality during patients’ in-hospital stay, specifically within the timeframe from admission to discharge. We focused on intracranial hemorrhage, composite complications, and death. We aimed to predict 6 outcomes: bleeding, composite complications, and all-cause death, both before and after thrombolytic therapy. Bleeding was identified from discharge diagnosis reports and head examination results, encompassing keywords and diagnostic conclusions such as hemorrhage and hematoma. Composite complications included reinfarction, cardiogenic shock, ischemic shock, and congestive heart failure, as extracted from the discharge diagnosis report. Death was defined by clear records of inpatient deaths during hospitalization, also sourced from discharge diagnosis reports.

### Feature selection and data preprocessing

We collected the patients’ basic information (sex and age), medical history (hypertension, atrial fibrillation, tumor, diabetes), vital signs (initial symptoms, heart rate, blood oxygen saturation, blood pressure, and respiration), clinical tests (liver and kidney function, blood sugar, electrolytes, and myocardial infarction indicators), and treatment records (hospital delay time, medication, and onset time). Common and cardiovascular-specific variables were structured and manually normalized independently by 2 professional doctors and subjected to consistency checks. For the 595 features obtained, the indicators were divided into pre- and post-thrombolytic therapy categories.

Variables with >30% missing values were excluded. Subsequently, 69 variables related to the initial hospitalization were extracted. Obvious outliers were replaced with null values, binary variables with consistency >90%, and continuous variables with variance <0.05 were deleted [50]. We validated the model performance according to different variance thresholds and found that the model retained a high and stable performance around the threshold of 0 to 1, which indicates that the level of 0.05 is reasonable (Fig. S6). Ten discrete and 27 continuous variables were retained. Bayesian and LR classifiers were used to interpolate the missing values for continuous and discrete random variables, respectively. Following interpolation, the Spearman’s correlation coefficient between each pair of 37 variables was calculated. For combinations with a correlation coefficient of 0.7 or higher, the variable that maximized the model’s AUC was retained, whereas the variable with a lower AUC was discarded. Two heatmaps are attached for comparison. One-hot encoding was performed on discrete variables, which were all binary, to avoid dimensionality issues. Ultimately, 31 features were collected, including sex, surgical history, past medical history, allergy history, familial clustering disease, smoking history, drinking history, β blockers, statins, age, pre-thrombolysis temperature, pre-thrombolysis systolic blood pressure, pre-thrombolysis pulse, pre-thrombolysis diastolic blood pressure, post-thrombolysis pulse, post-thrombolysis diastolic blood pressure, post-thrombolysis neutrophils (%), post-thrombolysis basophils count (#), post-thrombolysis eosinophils (%), post-thrombolysis urea, post-thrombolysis uric acid, post-thrombolysis mean corpuscular volume, post-thrombolysis total cholesterol, post-thrombolysis total protein, post-thrombolysis lymphocytes (#), post-thrombolysis albumin, post-thrombolysis creatinine, post-thrombolysis platelet (#), post-thrombolysis hemoglobin, in-hospital delay time, and onset time; 14 features were from the pre-thrombolysis period (Fig. S7 and Table S4).

### Machine learning model development and performance evaluation

This study was structured into 2 stages, pre-thrombolysis and post-thrombolysis, with each stage further categorized into 3 outcomes: bleeding, compound complications, and death. In the first stage, we employed 5 major models, the LR, DT, RF, XGBoost, and LightGBM models, to train and validate the 14 pre-thrombolysis indicators. The optimal model for each group was selected based on the highest AUC. Subsequently, we identified the top 8 optimal indicators through radar chart analysis. In the second stage, we continued with these 5 major models and incorporated post-thrombolysis indicators. The models were trained and validated using all included indicators before and after thrombolysis, with the optimal model selected based on the highest AUC value. Similarly, the top 8 optimal indicators were identified through radar chart analysis.

The datasets were divided into training (70%) and validation (30%). Five approaches were explored using 5-fold cross-validation to build the 5 models and adjust the parameters. As described in previous studies [51,52], the synthetic minority oversampling technique was performed solely on the training sets for the 3 outcomes to address imbalanced data issues. A random search and 5-fold cross-validation were used to optimize the hyperparameters and maximize the AUC. Fifteen models were developed separately for each outcome. The validation dataset was then used to evaluate the model performance metrics and identify the optimal model.

### Statistical analysis

Categorical variables were presented as percentages, whereas continuous variables are presented as mean [standard deviation (SD)] or median [interquartile range (IQR)]. Machine learning algorithms were implemented using the scikit-learn (version 0.21.1) package in Python (version 3.9.6; Python Software Foundation), and statistical analysis was conducted using the open-source SciPy (version 1.3.0) database from Python (version 3.9.6). Evaluation metrics, such as the AUC of the ROC curve, accuracy, precision, and recall, were analyzed to evaluate the model performance. In our study, we focused more on the ability of the model to assess the aforementioned events, which have greater clinical significance. Therefore, AUC, an indicator of the comprehensive performance of the model, is of paramount importance [53,54].

## Data Availability

The Python scripts related to this study are available at https://doi.org/10.5281/zenodo.13999336. Interested readers can request the test data from Tongji Hospital from our corresponding author. Data use will be restricted to noncommercial research purposes and must comply with the relevant laws and regulations of China. All authors who use the data or information from this study should refer to this paper.
