# Supplementary material for: An Explainable Two-Stage Machine Learning Model for Predicting the Post-Thrombolysis Complications in Stroke Patients: A Multi-Center Study
Source: Research (Wash D C). 2025 Aug 19;8:0817. doi: 10.34133/research.0817 (PMC12364525; doi:10.34133/research.0817)
Supplement: Supplementary 1 — Figs. S1 to S7 Tables S1 to S4 [file research.0817.f1.docx]

**Supplementary Information**

**Supplementary Table 1:** **Clinical features of the patients in external validation datasets. Data are showed as n (%), or n/N (%), and medium (IQR) where N is the total number of patients with available data.**

|  | **YX** | **XT** |
| --- | --- | --- |
| **Demographic information** | | |
| Age (years) | 67(57.5-74) | 70(63-75) |
| Sex (Male) | 94(69.63%) | 239(61.13%) |
| **Comorbidities** | | |
| Coronary heart disease | 11(8.15%) | 73(18.67%) |
| High blood pressure | 82(60.74%) | 228(58.31%) |
| Diabetes | 13(9.63%) | 66(16.88%) |
| Hyperlipidemia | 32(23.70%) | 13(3.32%) |
| Cerebrovascular diseases | 24(17.78%) | 75(19.18%) |
| Pregnant history | 0(0%) | 5(1.28%) |
| Surgical history | 14(10.37%) | 70(17.90%) |
| Familial clustering disease | 1(0.74%) | 1(0.26%) |
| Allergy history | 0(0%) | 2(0.51%) |
| smoking history | 23(17.04%) | 121(30.95%) |
| Drinking history | 13(9.63%) | 82(20.97%) |
| **Timing** | | |
| Onset time | 180(117.6-240) | 120(90-180) |
| In-hospital delay time | 31.8(1.2-75.6) | 64.2(54-87.6) |
| **thrombolytic agent** | | |
| Urokinase | 2(1.48%) | 94(24.04%) |
| Fibrinolysin | 0 | 0 |
| Alteplase | 104(77.04%) | 238(60.87%) |
| Reteplase | 0 | 0 |
| Recombinant Streptokinase | 0 | 0 |
| Tenecteplase | 29(21.48%) | 59(15.09%) |
| **Pre-thrombolysis Vital signs** | | |
| Diastolic blood pressure | 85(75.5-93.5) | 88(80-95) |
| Systolic blood pressure | 146(133.5-158) | 152(137-165) |
| Respiratory rate | 20(20-20) | 20(20-20) |
| Temperature | 36.42(36.3-36.5) | 36.5(36.35-36.7) |
| **Post-thrombolysis Vital signs** | | |
| Diastolic blood pressure | 84(75-90) | 86(79-94) |
| Systolic blood pressure | 142(129-155) | 150(136-160) |
| Respiratory rate | 20(20-20) | 20(20-20) |
| Temperature | 36.5(36.3-36.6) | 36.5(36.4-36.7) |
| **Medication use during hospitalization** | | |
| ACEI | 0(0%) | 37(9.46%) |
| ARB | 4(2.96%) | 41(10.49%) |
| β blocker | 6(4.44%) | 31(7.93%) |
| Statin | 89(65.93%) | 80(20.46%) |
| Clopidogrel | 61(45.19%) | 106(27.11%) |
| Aspirin | 65(48.15%) | 11(2.81%) |
| **Outcomes** | | |
| Death | 2(1.48%) | 4(1.02%) |
| compound complications events | 10(7.41%) | 1(0.26%) |
| Bleeding | 6(4.44%) | 86(21.99%) |

### **Supplementary Table 2: Model performance of bleeding, composite complications and death with 5 models on datasets from Tongji Hospital.**

| **Bleeding** | **Model** | **AUC**  **(95% CI)** | **Accuracy**  **(95% CI)** | **Precision**  **(95% CI)** | **Specificity**  **(95% CI)** | **Sensitivity**  **(95% CI)** | **F1**  **(95% CI)** |
| --- | --- | --- | --- | --- | --- | --- | --- |
| **Pre-thrombolysis** | LR | 0.7234  (0.6527,0.7909) | 0.7048  (0.6767,0.7320) | 0.1107  (0.0800,0.1472) | 0.7119  (0.6832,0.7388) | 0.5915  (0.4643,0.7167) | 0.1866  (0.1362,0.2414) |
|  | DT | 0.6263  (0.5492,0.6983) | 0.8472  (0.8257,0.8688) | 0.1301  (0.0733,0.1912) | 0.8805  (0.8614,0.9000) | 0.2951  (0.1754,0.4238) | 0.18  (0.1058,0.2553) |
|  | RF | 0.6958  (0.6269,0.7607) | 0.896  (0.8782,0.9119) | 0.1959  (0.1150,0.2907) | 0.9347  (0.9181,0.9486) | 0.2612  (0.1567,0.3803) | 0.2239  (0.1345,0.3185) |
|  | XGBoost | 0.6568  (0.5843,0.7351) | 0.9325  (0.9175,0.9466) | 0.3243  (0.1714,0.4828) | 0.9774  (0.9683,0.9861) | 0.1774  (0.0909,0.2910) | 0.2299  (0.1190,0.3410) |
|  | LightGBM | 0.6412  (0.5649,0.7093) | 0.9325  (0.9175,0.9475) | 0.3333  (0.1915,0.5200) | 0.9772  (0.9673,0.9859) | 0.1935  (0.1060,0.2969) | 0.2449  (0.1429,0.3636) |
| **Post-thrombolysis** | LR | 0.7469  (0.6767,0.8074) | 0.702  (0.6729,0.7252) | 0.1088  (0.0767,0.1417) | 0.7085  (0.6795,0.7351) | 0.5932  (0.4630,0.7111) | 0.184  (0.1337,0.2347) |
|  | DT | 0.659  (0.5780,0.7336) | 0.7076  (0.6785,0.7338) | 0.0988  (0.0654,0.1331) | 0.7197  (0.6920,0.7460) | 0.5079  (0.3793,0.625) | 0.1658  (0.1127,0.2177) |
|  | RF | 0.7581  (0.6955,0.8177) | 0.9157  (0.8978,0.9316) | 0.2647  (0.1666,0.3696) | 0.9533  (0.9396,0.9665) | 0.2797  (0.1764,0.3913) | 0.2731  (0.1724,0.3670) |
|  | XGBoost | 0.7553  (0.6837,0.8197) | 0.9419  (0.9278,0.9560) | 0.4857  (0.2916,0.6667) | 0.9861  (0.9790,0.9930) | 0.2143  (0.1186,0.3279) | 0.2963  (0.1728,0.4186) |
|  | LightGBM | 0.7484  (0.6769,0.8137) | 0.9428  (0.9278,0.9569) | 0.5  (0.2903,0.6875) | 0.9861  (0.9782,0.9929) | 0.2281  (0.1250,0.3463) | 0.3125  (0.1842,0.4421) |

| **Composite complications** | **Model** | **AUC**  **(95% CI)** | **Accuracy**  **(95% CI)** | **Precision**  **(95% CI)** | **Specificity**  **(95% CI)** | **Sensitivity**  **(95% CI)** | **F1**  **(95% CI)** |
| --- | --- | --- | --- | --- | --- | --- | --- |
| **Pre-thrombolysis** | LR | 0.6497(0.6164,0.6841) | 0.5689(0.5389,0.5979) | 0.3603(0.3207,0.3993) | 0.5306(0.4939,0.5658) | 0.6655(0.6109,0.7183) | 0.4672(0.4259,0.5063) |
|  | DT | 0.6517(0.6148,0.6906) | 0.6617(0.6326.0.6898) | 0.4266(0.3768,0.4740) | 0.7027(0.6710,0.7357) | 0.5588(0.5051,0.6141) | 0.4830(0.4394,0.5273) |
|  | RF | 0.7035(0.6685,0.7392) | 0.7291(0.7038,0.7545) | 0.5244(0.4640,0.5787) | 0.8404(0.8153,0.8646) | 0.4466(0.3947,0.5000) | 0.4828(0.4340,0.5271) |
|  | XGBoost | 0.6743(0.6358,0.7106) | 0.7226(0.6935,0.7470) | 0.5100(0.4449,0.5782) | 0.8717(0.8478,0.8946) | 0.3426(0.2885,0.3943) | 0.4093(0.3539,0.4622) |
|  | LightGBM | 0.6718(0.6381,0.7072) | 0.7226(0.6973,0.7479) | 0.5146(0.4412,0.5902) | 0.8887(0.8669,0.9102) | 0.2997(0.2508,0.3500) | 0.3790(0.3279,0.4316) |
| **Post-thrombolysis** | LR | 0.6993(0.6682,0.7309) | 0.6279(0.6007,0.6551) | 0.4082(0.3640,0.4473) | 0.5966(0.5619,0.6274) | 0.7083(0.6598,0.7565) | 0.5183(0.4765,0.5556) |
|  | DT | 0.6942(0.6599,0.7287) | 0.6935(0.6664,0.7226) | 0.4665(0.4130,0.5149) | 0.7510(0.7175,0.7824) | 0.5517(0.5000,0.6087) | 0.5038(0.4571,0.5510) |
|  | RF | 0.7584(0.7299,0.7901) | 0.7404(0.7151,0.7667) | 0.5546(0.4841,0.6177) | 0.8655(0.8401,0.8906) | 0.4224(0.3636,0.4795) | 0.4789(0.42552,0.5316) |
|  | XGBoost | 0.7602(0.7299,0.7907) | 0.7535(0.7291,0.7798) | 0.6117(0.5404,0.6864) | 0.9113(0.8920,0.9304) | 0.3540(0.3049,0.4089) | 0.4478(0.3973,0.5010) |
|  | LightGBM | 0.7625(0.7324,0.7936) | 0.7441(0.7170,0.7685) | 0.5543(0.4941,0.6157) | 0.8524(0.8278,0.8777) | 0.4660(0.4080,0.5232) | 0.5065(0.4544,0.5550) |

| **Death** | **Model** | **AUC**  **(95% CI)** | **Accuracy**  **(95% CI)** | **Precision**  **(95% CI)** | **Specificity**  **(95% CI)** | **Sensitivity**  **(95% CI)** | **F1**  **(95% CI)** |
| --- | --- | --- | --- | --- | --- | --- | --- |
| **Pre-thrombolysis** | LR | 0.7097  (0.5597,0.8514) | 0.7657  (0.7385, 0.7901) | 0.0271  (0.0083,0.0496) | 0.7684  (0.7415,0.7923) | 0.5385  (0.2308,0.8) | 0.0516  (0.0161,0.0922) |
|  | DT | 0.7465  (0.5244,0.9278) | 0.8763  (0.8557,0.8950) | 0.0576  (0.0244,0.1029) | 0.8795  (0.8597,0.8985) | 0.6154  (0.3525,0.875) | 0.1046  (0.0454,0.1805) |
|  | RF | 0.8450  (0.7454,0.9375) | 0.8351  (0.8135,0.8566) | 0.0479  (0.0220,0.0833) | 0.8371  (0.8159,0.8585) | 0.6923  (0.4375,0.9375) | 0.0892  (0.0421,0.1500) |
|  | XGBoost | 0.7681  (0.6345,0.8891) | 0.9700  (0.9597,0.9794) | 0.0833  (0,0.2107) | 0.9801  (0.9715,0.9877) | 0.1429  (0,0.385) | 0.1053  (0,0.2564) |
|  | LightGBM | 0.7673  (0.5868,0.9307) | 0.9381  (0.9231,0.9522) | 0.0758  (0.0161,0.1526) | 0.9449  (0.9299,0.9583) | 0.3750  (0.1,0.6667) | 0.1250  (0.0282,0.2400) |
| **Post-thrombolysis** | LR | 0.8307  (0.7420,0.9089) | 0.8950  (0.8772,0.9129) | 0.0522  (0.0171,0.0990) | 0.9003  (0.8831,0.9183) | 0.4545  (0.1995,0.75) | 0.0930  (0.0317,0.1691) |
|  | DT | 0.7955  (0.6257,0.9344) | 0.9428  (0.9288,0.9560) | 0.1126  (0.0417,0.1967) | 0.9478  (0.9328,0.9611) | 0.5385  (0.2662,0.8462) | 0.1853  (0.0706,0.3038) |
|  | RF | 0.9264  (0.8736,0.9660) | 0.8491  (0.8266,0.8697) | 0.0699  (0.0341,0.1124) | 0.8480  (0.8256,0.8680) | 0.9286  (0.75,1) | 0.1297  (0.0658,0.2010) |
|  | XGBoost | 0.9005  (0.8209,0.9630) | 0.9841  (0.9766,0.9906) | 0.2500  (0,0.6364) | 0.9943  (0.9895,0.9981) | 0.1429  (0,0.375) | 0.1818  (0,0.4286) |
|  | LightGBM | 0.8807  (0.8032,0.9379) | 0.9691  (0.9578,0.9794) | 0.1053  (0,0.25) | 0.9782  (0.9687,0.9867) | 0.2143  (0,0.5) | 0.1395  (0,0.3077) |

## **Supplementary Figure 1: AUROC curves of bleeding, composite complications and death pre-thrombolysis, and post-thrombolysis using five machine learning models on Tongji Hospital dataset.** Bleeding with LR (A1), DT (A2), RF (A3), XGBoost (A4) and LightGBM (A5), composite complications with LR (B1), DT (B2), RF (B3), XGBoost (B4) and LightGBM (B5), death with LR (C1), DT (C2), RF (C3), XGBoost (C4) and LightGBM (C5), Pre-thrombolysis (red line), Post-thrombolysis (Blue line).


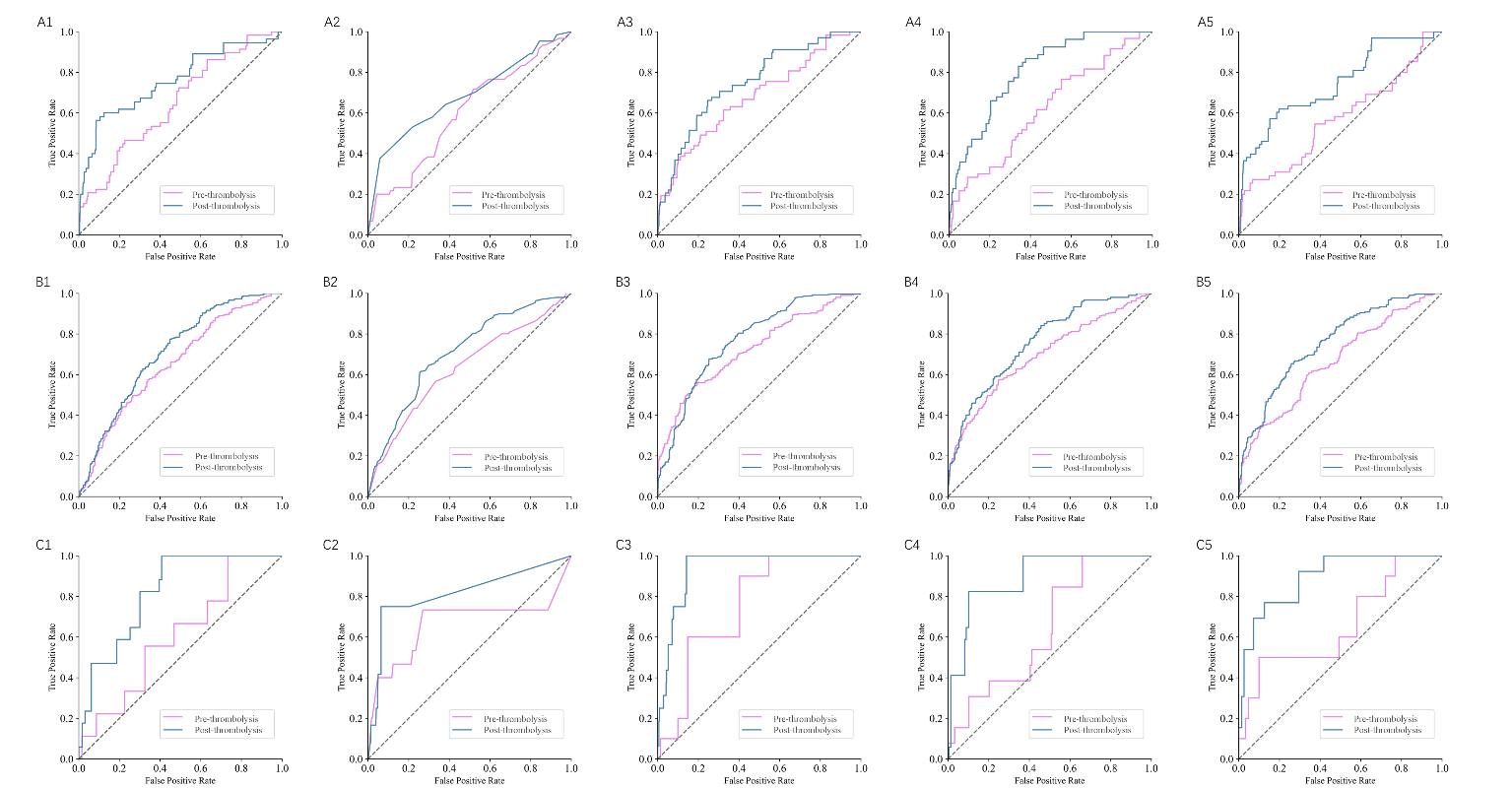


## **Supplementary Table 3: Performance summary of on the external datasets of YX dataset and XT dataset.**

| **YX dataset** | **Model** | **AUC**  **(95% CI)** | **Accuracy**  **(95% CI)** | **Precision**  **(95% CI)** | **Specificity**  **(95% CI)** | **Sensitivity**  **(95% CI)** | **F1**  **(95% CI)** |
| --- | --- | --- | --- | --- | --- | --- | --- |
| **Bleeding** | | | | | | | |
| **Pre** | LR | 0.3654 | 0.4412 | 0.0395 | 0.4385 | 0.5 | 0.0732 |
| **Post** | RF | 0.5462 | 0.9559 | 0 | 1 | 0 | 0 |
| **Composite complications** | | | | | | | |
| **Pre** | RF | 0.3365 | 0.9191 | 0 | 0 | 0.9921 | 0 |
| **Post** | LightGBM | 0.3111 | 0.9265 | 0 | 1 | 0 | 0 |
| **Death** | | | | | | | |
| **Pre** | RF | 0.6269 | 0.3382 | 0.0217 | 0.3284 | 1 | 0.0426 |
| **Post** | RF | 0.7724 | 0.6691 | 0.0222 | 0.6716 | 0.5 | 0.0426 |

| **XT dataset** | **Model** | **AUC**  **(95% CI)** | **Accuracy**  **(95% CI)** | **Precision**  **(95% CI)** | **Specificity**  **(95% CI)** | **Sensitivity**  **(95% CI)** | **F1**  **(95% CI)** |
| --- | --- | --- | --- | --- | --- | --- | --- |
| **Bleeding** | | | | | | | |
| **Pre** | LR | 0.4955 | 0.3018 | 0.2192 | 0.1475 | 0.8488 | 0.3484 |
| **Post** | RF | 0.4355 | 0.7749 | 0 | 0.9934 | 0 | 0 |
| **Composite complications** | | | | | | | |
| **Pre** | RF | 0.3538 | 0.9974 | 0 | 1 | 0 | 0 |
| **Post** | LightGBM | 0.8179 | 0.9974 | 0 | 1 | 0 | 0 |
| **Death** | | | | | | | |
| Pre | RF | 0.6182 | 0.2839 | 0.0106 | 0.2791 | 0.75 | 0.021 |
| Post | RF | 0.6899 | 0.4373 | 0.0135 | 0.4341 | 0.75 | 0.0265 |

**Supplementary Figure 2. Recall (sensitivity) curves of the best-performed model of bleeding, composite complications and death pre-thrombolysis, and post-thrombolysis on Tongji Hospital dataset.** Bleeding pre-thrombolysis (A) and post-thrombolysis (B), composite complication pre-thrombolysis (C) and post-thrombolysis (D), and death pre-thrombolysis (E) and post-thrombolysis (F)


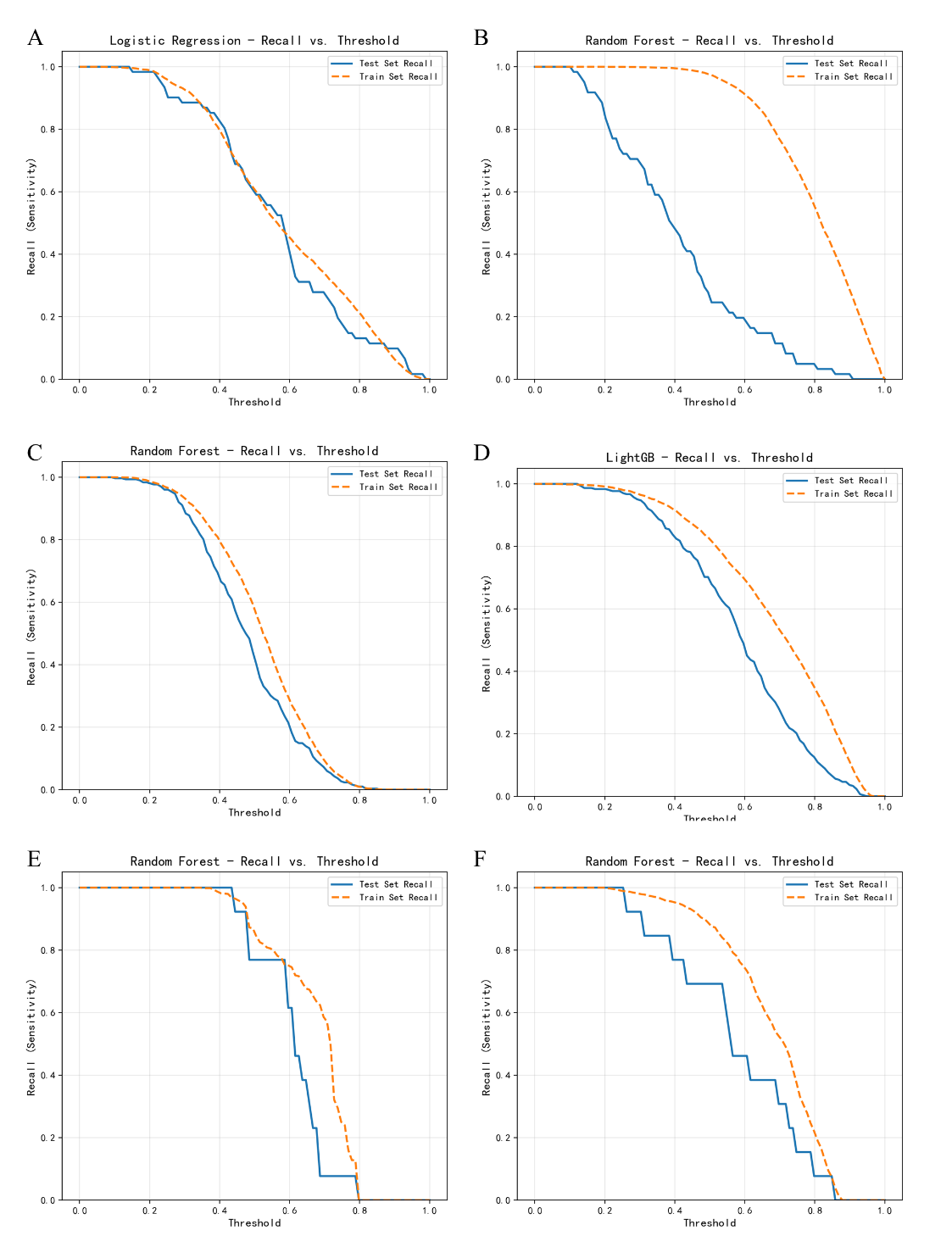


**Supplementary Figure 3. Calibration Curves of the best-performed model of bleeding, composite complications and death on Tongji Hospital dataset.** (*means pre-thrombolysis, ** means post-thrombolysis; Brown: bleeding, green: composite complications, blue: death)


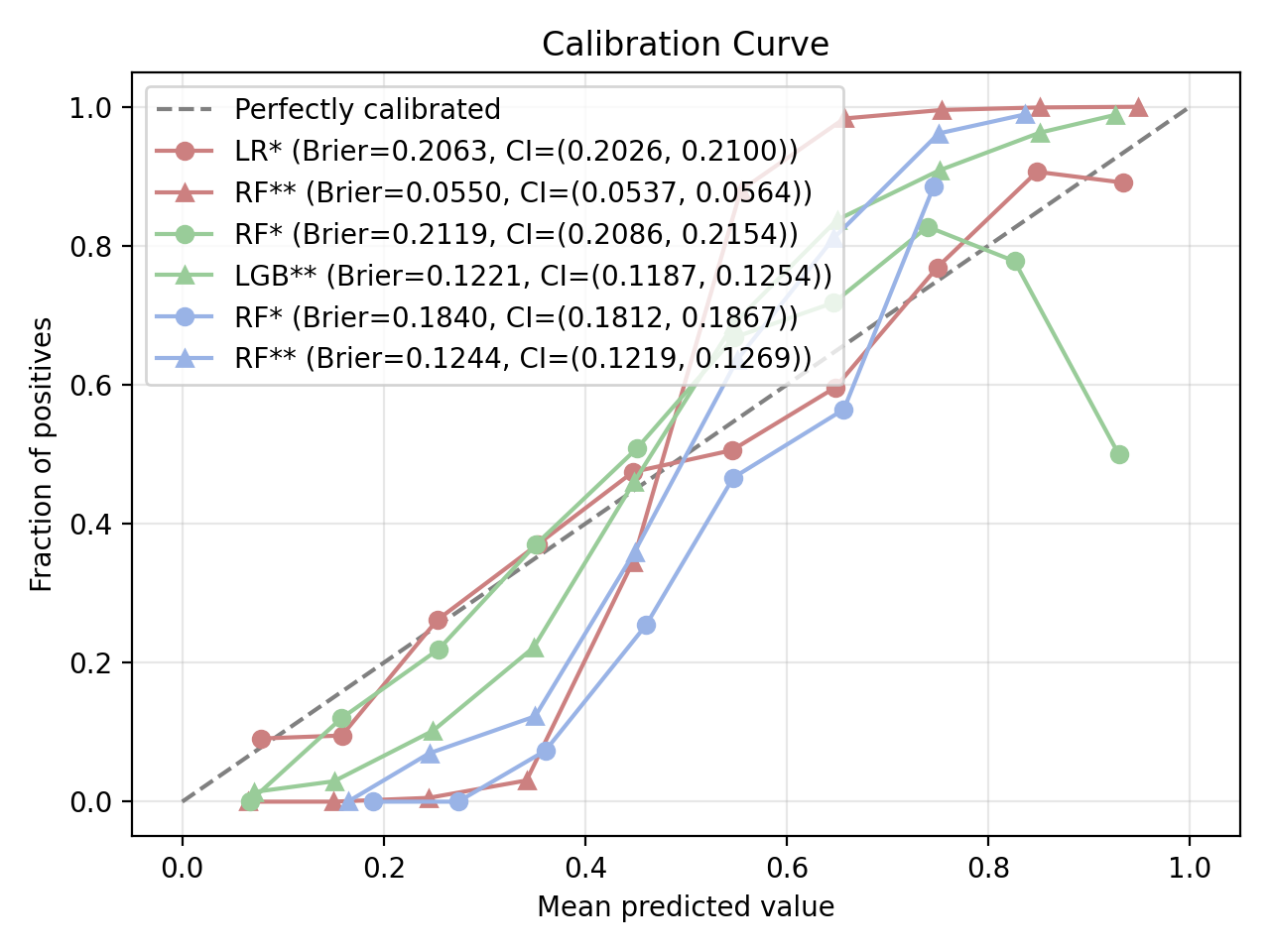


## **Supplementary Figure 4: AUROC curves of the best-performed model of bleeding, composite complications and death pre-thrombolysis, and post-thrombolysis on internal validation TJ-Test dataset.** Bleeding pre-thrombolysis (A) and post-thrombolysis (B), composite complication pre-thrombolysis (C) and post-thrombolysis (D), and death pre-thrombolysis (E) and post-thrombolysis (F)


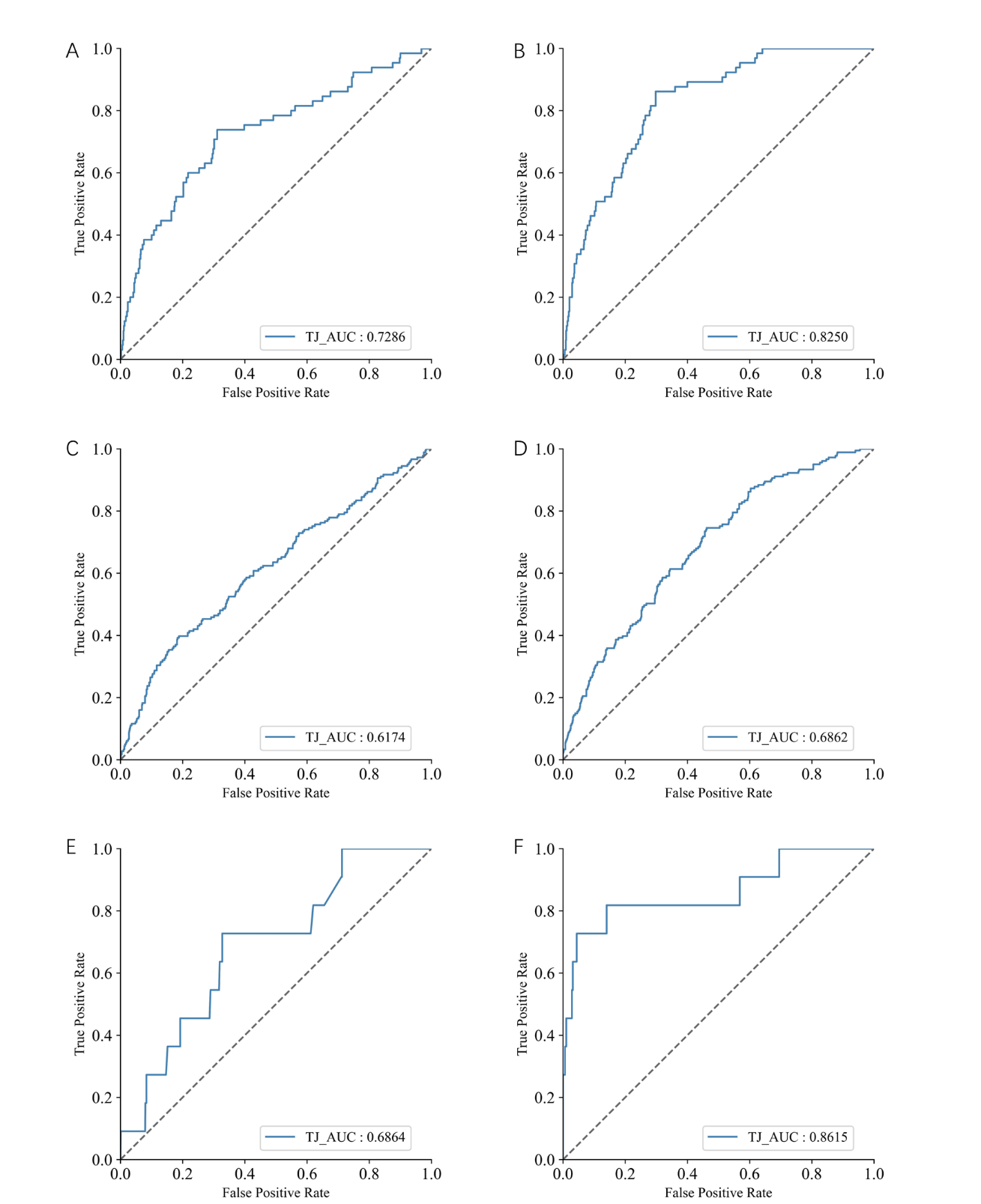


**Supplementary Figure 5: Radar plots for the eight most important predictors of bleeding, composite complications and death pre- and post-thrombolysis based on the best performed model on Tongji hospital dataset.** Bleeding pre-thrombolysis (A) and post-thrombolysis (B), composite complication pre-thrombolysis (C) and post-thrombolysis (D), and death pre-thrombolysis (E) and post-thrombolysis (F).

**
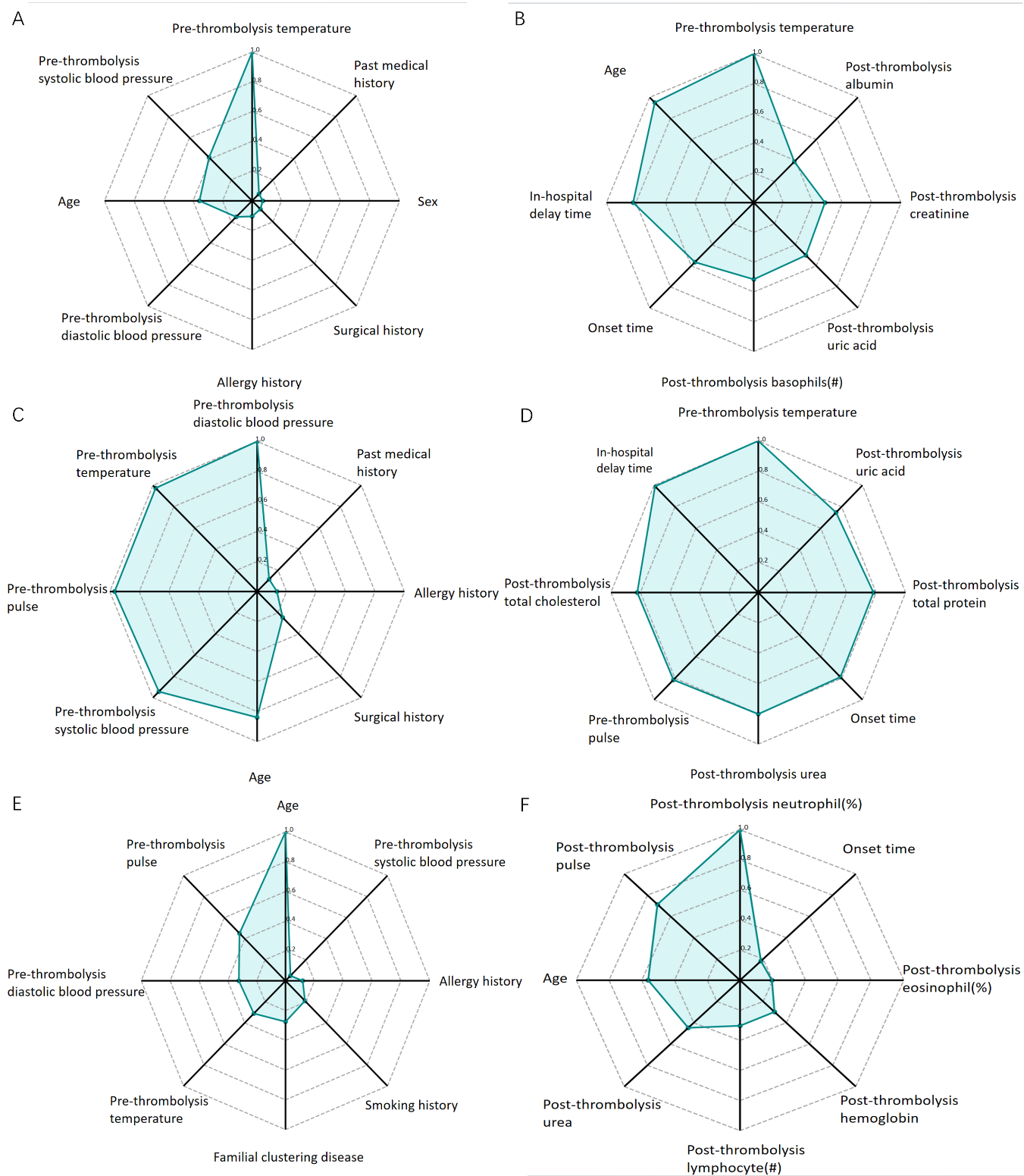
**

### **Supplementary Figure 6: The model performance according to different thresholds of feature filtering.**


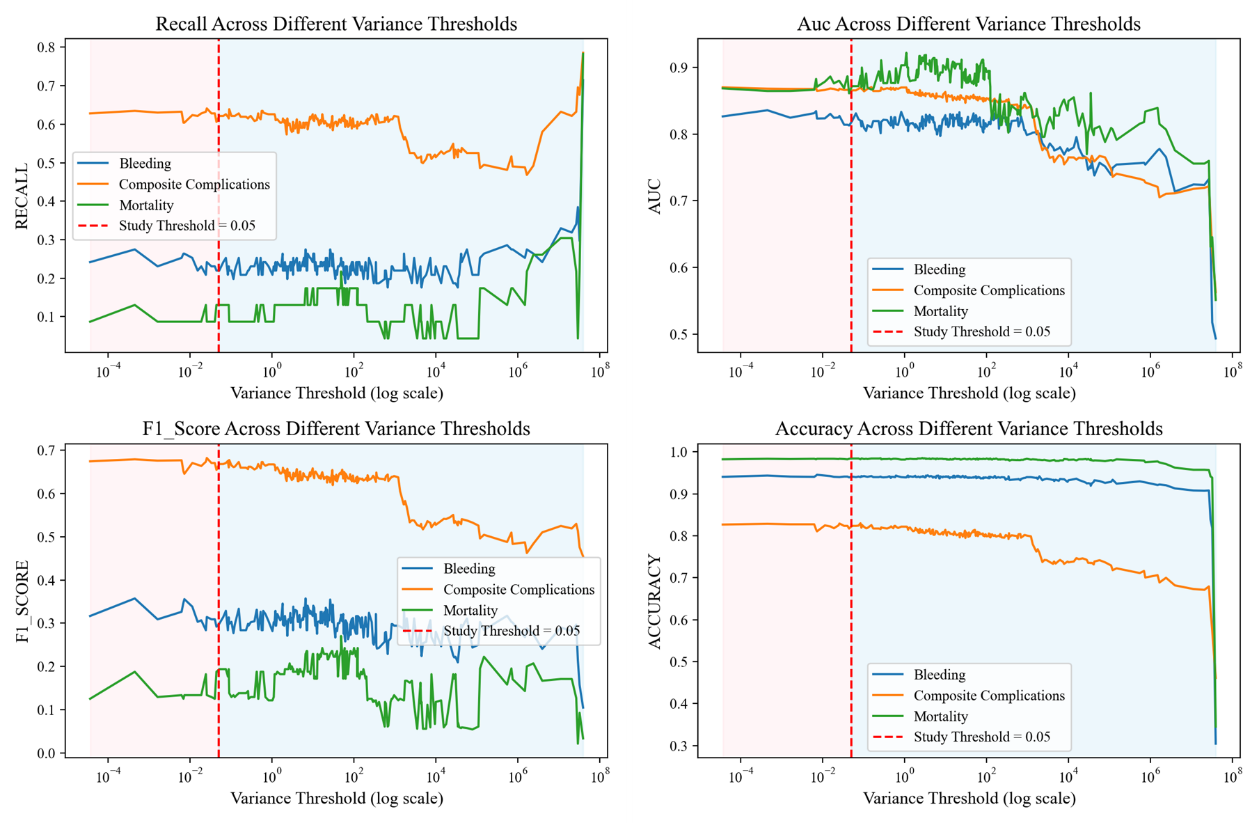


### **Supplementary Figure 7: The Spearman's correlation coefficient heatmap before (A) and after (B) feature selection.**

### **
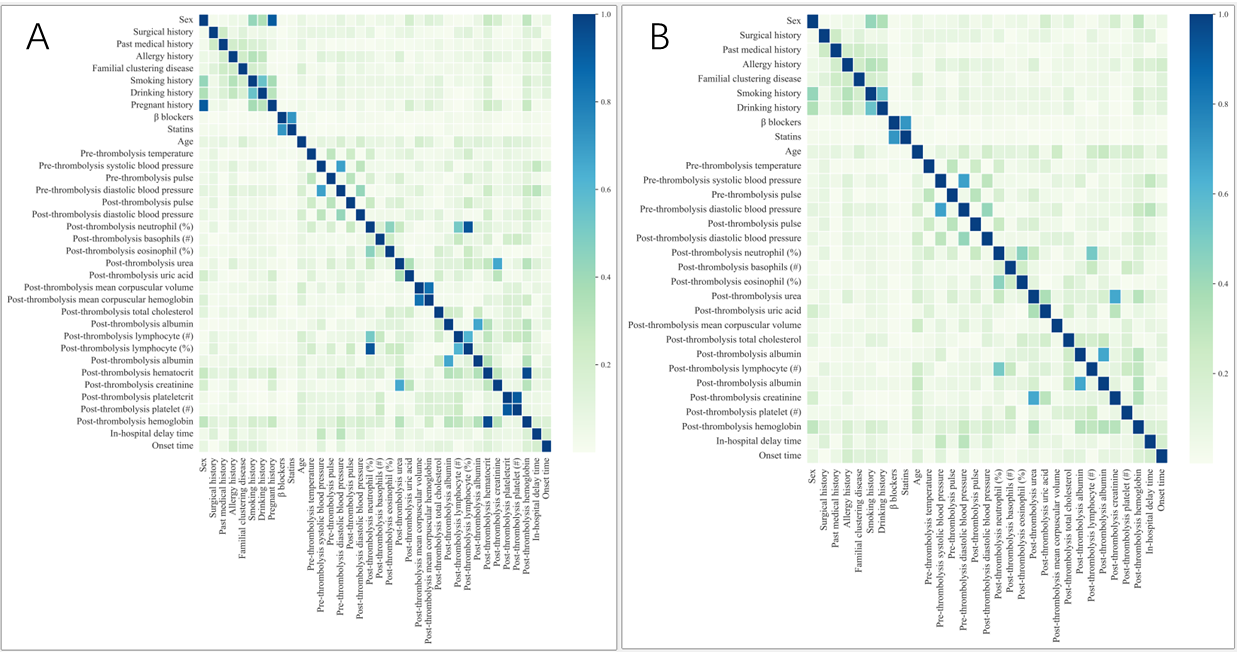
**

### **Supplementary Table 4: diagram of flowchat.**


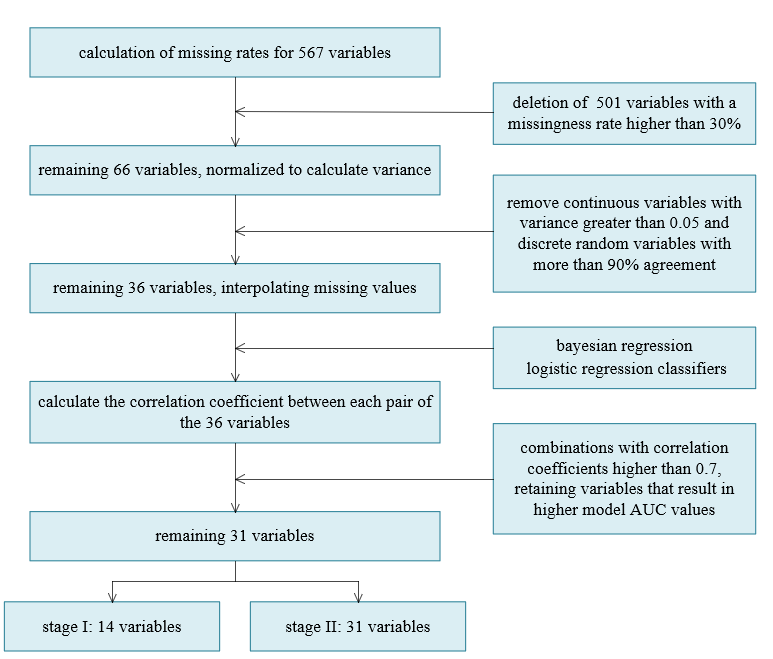


# **Statistical analysis details**

We use validation metrics to verify the performance of the proposed models. Subset accuracy is a strict evaluation metric, that is, the exact match ratio (EM), for this diagnosis problem according to the following definition:

$Subset accuracy/EM=\frac{1}{N}\sum_{n=1}^{N} \boldsymbol{1}_{\left\{ \tilde{Y} =Y \right\}}$ (10)

where $\boldsymbol{1}_{\left\{ \bar{Y}=Y \right\}}$ is the indicator function, and $N$ is the total number of samples to be assessed.

The validation metrics include AUROC, sensitivity, specificity, F1-score, and accuracy. These metrics can be calculated for each type of cases, each corresponding to a specific label $i$ as follows:

$\mathrm{Precisio}n_{i}=\frac{TP_{i}}{TP_{i}+FP_{i}}$ (12)

${\mathrm{Sensitivity}/\mathrm{Recall}}_{i}=\frac{TP_{i}}{TP_{i}+FN_{i}}$ (13)

$\mathrm{Specificity}_{i}=\frac{TN_{i}}{TN_{i}+FP_{i}}$ (14)

${F1}_{i}=\frac{2\times\mathrm{Precision}_{i}{\times Recall}_{i}}{\mathrm{Precision}_{i}+\mathrm{Recall}_{i}}$ (15)

where $TN_{i}$, $TP_{i}$, $\mathrm{FP}_{i}$, and $\mathrm{FN}_{i}$ are the true-positive, true-negative, false-positive, and false-negative rates for label $i$, respectively.
